# Multi-Modal Pain Intensity Assessment Based on Physiological Signals: A Deep Learning Perspective

**DOI:** 10.3389/fphys.2021.720464

**Published:** 2021-09-01

**Authors:** Patrick Thiam, Heinke Hihn, Daniel A. Braun, Hans A. Kestler, Friedhelm Schwenker

**Affiliations:** ^1^Institute of Medical Systems Biology, Ulm University, Ulm, Germany; ^2^Institute of Neural Information Processing, Ulm University, Ulm, Germany

**Keywords:** physiological signals, signal processing, deep neural networks, information fusion, pain intensity assessment

## Abstract

Traditional pain assessment approaches ranging from self-reporting methods, to observational scales, rely on the ability of an individual to accurately assess and successfully report observed or experienced pain episodes. Automatic pain assessment tools are therefore more than desirable in cases where this specific ability is negatively affected by various psycho-physiological dispositions, as well as distinct physical traits such as in the case of professional athletes, who usually have a higher pain tolerance as regular individuals. Hence, several approaches have been proposed during the past decades for the implementation of an autonomous and effective pain assessment system. These approaches range from more conventional supervised and semi-supervised learning techniques applied on a set of carefully hand-designed feature representations, to deep neural networks applied on preprocessed signals. Some of the most prominent advantages of deep neural networks are the ability to automatically learn relevant features, as well as the inherent adaptability of trained deep neural networks to related inference tasks. Yet, some significant drawbacks such as requiring large amounts of data to train deep models and over-fitting remain. Both of these problems are especially relevant in pain intensity assessment, where labeled data is scarce and generalization is of utmost importance. In the following work we address these shortcomings by introducing several novel multi-modal deep learning approaches (characterized by specific supervised, as well as self-supervised learning techniques) for the assessment of pain intensity based on measurable bio-physiological data. While the proposed supervised deep learning approach is able to attain state-of-the-art inference performances, our self-supervised approach is able to significantly improve the data efficiency of the proposed architecture by automatically generating physiological data and simultaneously performing a fine-tuning of the architecture, which has been previously trained on a significantly smaller amount of data.

## 1. Introduction

The area of research specific to the development of autonomous and objective pain assessment and management systems has been attracting a lot of interest from both medical and engineering research communities lately (Argüello Prada, 2020; Eccleston et al., 2020; Walter et al., 2020). This is due to the fact that an automatic and effective pain assessment system is more than desirable in the context of telemedicine and remote patient monitoring (Schobel et al., 2021), as well as in cases where an individual is unable to accurately assess and successfully report some currently experienced or observed pain episode. The inability to properly assess and effectively report specific pain episodes can be caused by various factors ranging from psychological or cognitive impairments, to physical and cultural predispositions. In such cases, the reliance on self-reporting tools such as the Visual Analogue Scale (VAS) (Hawker et al., 2011) or the Numerical Rating Scale (NRA) (Eckard et al., 2016) would potentially lead to some unsuitable and inadequate pain relief therapy. Meanwhile, suitable information stemming from an autonomous and objective pain assessment system based on measurable behavioral, anatomical and physiological parameters could provide some additional and relevant insight regarding the underlying pain episode, therefore helping to significantly improve both pain assessment and management.

In concordance with the aforementioned increasing interest, as well as technological advances in such areas as sensor systems and data persistence (which enables researchers to proceed with the recording of a diverse set of measurable autonomic parameters using a plethora of advanced sensor systems and wearables), a gradually growing amount of approaches are being proposed for the development of automatic pain assessment systems. Most of these approaches consist of various machine learning methods built upon different types of collected audiovisual and bio-physiological data, that are optimized and subsequently applied in both clinical and experimental settings. Depending on the amount and diversity of sensors used during the data collection phase, several signals have been assessed and evaluated in various settings for the development of pain assessment systems. Some of the most prominently used signals constitute of the audio signal (e.g., paralinguistic vocalizations) (Tsai et al., 2016, 2017; Thiam et al., 2017; Thiam and Schwenker, 2019), the video signal (e.g., facial expressions) (Rodriguez et al., 2017; Werner et al., 2017; Tavakolian and Hadid, 2019; Thiam et al., 2020b), specific bio-physiological signals such as the Electrodermal Activity (EDA), the Electrocardiogram (ECG), the Electromyography (EMG), or the Respiration (RSP) signal (Walter et al., 2014; Campbell et al., 2019; Thiam et al., 2019a), and also bodily expression signals (Dickey et al., 2002; Olugbade et al., 2019; Uddin and Canavan, 2020).

According to the variety of data collected, different types of machine learning approaches have also been proposed and assessed to perform a specific and effective pain assessment task. The proposed approaches range from uni-modal techniques which rely on a single modality (or channel) to perform the underlying inference task, to multi-modal techniques which rely on a set of multiple and diverse modalities to perform the underlying pain assessment task. Typical uni-modal approaches consist of extracting relevant information in the form of a specific feature representation from the underlying modality and subsequently using the feature representation to perform the optimization of a specific inference model (Sharma et al., 2019, 2020). Multi-modal approaches on the other hand, are designed to perform an aggregation of a set of information stemming from multiple and heterogeneous modalities by applying a specific information fusion technique, in order to improve both the performance as well as the robustness of an inference system. Rather than relying on a single channel, an effective and smart combination of complementary information stemming from multiple channels mitigates the drawbacks specific to each single channel, while improving the generalization ability of the optimized inference system in comparison to one based on a single modality (Kächele et al., 2016; Bellmann et al., 2018; Thiam et al., 2018).

In the following work, a multi-modal information aggregation approach based on Deep Denoising Convolutional Auto-Encoders (DDCAEs) is proposed for the assessment of pain intensities based on bio-physiological signals, and subsequently evaluated in terms of classification, regression and data efficiency performances. The proposed approach is characterized by a concurrent and autonomous optimization of the feature representations specific to the involved channels, as well as the simultaneous optimization of a feed-forward neural network performing the underlying inference task. The contribution of the current work is four-fold: first of all, a multi-modal DDCAE architecture (originally proposed in Thiam et al., 2020a) is proposed and described, for the assessment of different levels of pain elicitation based on a set of diverse bio-physiological modalities. Secondly, a gating layer is also proposed and used in combination with the multi-modal DDCAE architecture in order to perform the aggregation of the information stemming from the underlying modalities before being subsequently used to perform a specific inference task. The resulting architecture is consistently evaluated and further extended with an attention mechanism in order to significantly improve the performance of the designed inference system. Next, the resulting model is further extended by introducing a novel Self-Supervised Learning approach to improve the data efficiency of the designed deep architecture. Self-Supervised Learning (SSL) (Jing and Tian, 2020; Jaiswal et al., 2021) is a form of representation learning (Bengio et al., 2013), where the aim is to learn a meaningful representation to improve a final supervised learning task. Our SSL method is based on an information-theoretic approach which enables Variational Auto-Encoders (VAEs) (Kingma and Welling, 2014) to learn a meaningful and compressed data representation. This representation is then used to generate new data which in turn is utilized to perform a final fine-tuning step. To further improve the generalization ability of the deep model, we apply an information-processing constraint on the Auto-Encoders, the fusion gate, and on the classifier. A consistent benchmark of the designed approaches is provided based on both the BioVid Heat Pain Database (Walter et al., 2013) and the SenseEmotion Database (Velana et al., 2017), where we were able to show that our SSL approach produces results that are only marginally lower compared to data augmentation while only using a fraction of the data.

The remainder of the present work is structured as follows. Section 2 provides an overview of some related work involving multi-modal approaches for pain assessment based on bio-physiological signals. This includes conventional as well as deep learning approaches. The proposed approaches, as well as the data used for the assessment of the proposed approaches are described in section 3. The results specific to the performed experiments are depicted and described in section 4. Finally, a discussion of the achieved results, as well as a description of potential future works is provided in section 5, and the work is concluded with an outlook in section 6.

## 2. Related Work

Multi-modal information fusion approaches are designed with the primary goal of significantly improving the performance of an inference model by effectively combining complementary information stemming from a set of diverse modalities (Kittler and Roli, 2000; Kuncheva, 2004; Palm and Schwenker, 2009; Roli, 2009). Conventional information fusion approaches therefore rely on a set of carefully designed hand-crafted features (extracted individually from each input channel) in combination with an information aggregation approach in order to perform the underlying inference task. Hence, the overall performance of the resulting inference model depends on both the relevance of the extracted feature representations (with regards to the underlying inference task), as well as the ability of the designed aggregation approach to effectively combine the information stemming from the resulting heterogeneous set of hand-crafted features. Some of the most prominently used fusion methods consist of early fusion and late fusion approaches.

Early fusion consists of concatenating the extracted feature representations specific to the underlying modalities into a single and high dimensional feature representation, which is subsequently fed into a classification (or regression) model in order to perform the corresponding inference task. The authors in Walter et al. (2014) extract various features from each input channel [EMG, ECG, Skin Conductance Level (SCL)] and perform the classification of several levels of heat-induced pain intensity using early fusion in combination with a Support Vector Machine (SVM) classification model (Abe, 2010). Similarly, the authors in Chu et al. (2014) extract features from similar modalities and use a combination of early fusion and Linear Discriminant Analysis (LDA) (Fisher, 1936) to perform the classification of several levels of electrical pain stimulation. In Chu et al. (2017), the authors also perform an early fusion of a set of features extracted individually from each modality including SCL, ECG, Blood Volume Pulse (BVP), and subsequently selected using genetic algorithms. The classification is subsequently performed using either a SVM, a k-Nearest Neighbor (k-NN) algorithm or a LDA model. The authors in Werner et al. (2014) and Kächele et al. (2015) extract various features from each input channel and perform the classification of several levels of heat-induced pain intensity using early fusion in combination with a Random Forest (RF) classification model (Breiman, 2001). In Ricken et al. (2020), the authors use the same approach in order to perform the classification of different levels of thermal and electrical pain stimuli, based on a similar set of modalities.

Late fusion on the other hand, consists of the combination at a higher level of aggregation of the outputs of a diverse set of inference models trained on various feature representations. In Kessler et al. (2017), the authors designed and assessed an hierarchical fusion architecture consisting of an Artificial Neural Network (ANN) and the Moore-Penrose Pseudoinverse aggregation rule (Schwenker et al., 2006) for the aggregation of several base classifiers' outputs (consisting of RF models), in order to perform the classification of several levels of artificially induced pain elicitation based on several bio-physiological signals including remote Photoplethysmography (rPPG), ECG, and RSP. In Bellmann et al. (2020), the authors propose a dominant channel fusion approach consisting of first identifying the most relevant input channel and using a subsequent combination of the identified most relevant channel and the remaining ones to create an ensemble of classifiers. The final output of the resulting ensemble is computed by applying an average (Mean) aggregation rule. The approach is assessed on several data sets comprising bio-physiological modalities such as EMG, ECG, EDA, and RSP. In Bellmann et al. (2021), a novel late fusion approach consisting of a combination of mixture of experts and stacked generalization approaches is proposed and assessed on different data sets involving the bio-physiological modalities EMG, ECG, and EDA. The authors in Kächele et al. (2016), Thiam and Schwenker (2017), and Werner et al. (2019) use a combination of RF classification models (trained individually on various feature representations), and a Moore-Penrose Pseudoinverse aggregation approach in order to perform the underlying pain related classification tasks. In Lim et al. (2019), the authors propose a bagged ensemble of Deep Belief Networks (DBNs) (Lopes and Ribeiro, 2015) for the assessment of patient's pain level during surgery, using photoplethysmography (PPG). The ensemble of bagged DBNs is also trained on a set of handcrafted features.

Meanwhile, the processes involved in the manual engineering of feature representations and the selection of relevant features for a specific modality are complex and time consuming. Some specific expert knowledge in the area of application is needed in order to ensure that the resulting and final feature representation is relevant for the task at hand. Moreover, since each single feature representation is specific to the corresponding channel and generated independently from the other task-related modalities, finding a suitable information aggregation approach, that effectively combines the complementary information stemming from the channels, can be very tedious. Thus, a growing amount of work has been investigating the application of deep learning approaches with the goal of enabling a system to autonomously learn not only suitable feature representations, but also effective information aggregation parameters, directly from the corresponding and preprocessed raw input signals. The authors in Thiam et al. (2019a) propose a deep neural network for the classification of different levels of nociceptive pain based on ECG, EMG, and EDA signals, characterized by a weighted aggregation layer performing the combination of the outputs of modality specific Convolutional Neural Networks (CNNs) (LeCun et al., 2015). The whole architecture is trained in an end-to-end manner and was able to attain state-of-the-art classification performances on the BioVid Heat Pain Database. In Thiam et al. (2020a), the authors perform a benchmarking of different types of multi-modal DDCAE architectures, each model characterized by a specific joint representation learned simultaneously from various input modalities. In Subramaniam and Dass (2021), the authors propose a hybrid deep learning network consisting of shallow CNNs that extract information from the raw input signals, and the resulting feature representations are subsequently fed to a Long Short-Term Memory (LSTM) recurrent neural network (Hochreiter and Schmidhuber, 1997) that performs the aggregation of the extracted information followed by the classification of different levels of pain elicitation. The approach is also evaluated on The BioVid Heat Pain Database, with the ECG and EDA modalities as input signals.

Self-Supervised Learning has seen some recent attention in the machine learning community. Most algorithms and approaches fall into the category of image classification and generation (Tung et al., 2017) and language modeling (Lan et al., 2020), but the principle is general enough to be applied to a variety of learning problems (Baevski et al., 2020; Ravanelli et al., 2020; Sekar et al., 2020). Generative models [e.g., Generative Adverserial Nets (Goodfellow et al., 2014), Variational Auto-Encoders (Kingma and Welling, 2014), and Boltzmann Machines (Salakhutdinov and Hinton, 2009)] have been recently successfully applied to learn feature representations (Pathak et al., 2016; Donahue et al., 2017; Zhang et al., 2017), as generative models are a natural example of SSL models as the main goal is to find a representation that produces realistic data, e.g., photo-realistic images. However, there has been only little prior work that utilizes SSL in the context of automatic pain assessment and related fields. The work of Tavakolian et al. (2020) proposes a SSL approach to facial recognition for automatic pain assessment. They implement a novel similarity metric and train a Siamese Network on video streams to optimize the metric, distill the network, and then fine-tune the network on pain assessment training data. The authors of Das et al. (2021) introduce an explainable Self-Supervised Representation Learning paradigm to learn temporal facial patterns. They apply their method to predict speech behavior from stuttering adults. Both methods we discussed are specific to their application, whereas our SSL method is general enough to be applied to any learning problem. At the time of publication of this study there were no further SSL applications to automatic pain assessment to the best of our knowledge.

The information-theoretic SSL principle we propose is based on two main ideas: (i) variational Auto-Encoders with adaptive priors and (ii) a encoder-decoder hierarchy. Adaptive VAEs have been first introduced by Hihn et al. (2018) as a way to learn a data dependent prior that can be use to generate new samples efficiently. Although evaluated only on small scale data it has shown promising results, as the introduced information processing constraint enforces abstract latent representations retaining only information that is useful for the task at hand. The idea of using information processing constraints has been investigated extensively in the reinforcement learning community (Houthooft et al., 2016; Galashov et al., 2019; Grau-Moya et al., 2019; Hihn et al., 2019; Leibfried et al., 2019). We extend this idea to the supervised learning setting and combine it further with a encoder-decoder structure used for classification. In Peng et al. (2017), the authors introduce an information-theoretic formalization of a encoder-decoder hierarchy that is based on bounded rationality (Genewein et al., 2015). The authors show how such policies can be learned in an on-line manner. They evaluate their method in a reinforcement learning setting using a simulated humanoid robot platform and show that it is able to learn a meaningful representation of the environment.

The current work aims at improving the performance of a pain assessment model by enabling a specific ANN to perform autonomously and simultaneously both the extraction of feature representations specific to the input modalities, as well as the aggregation of the information stemming from the generated representations. Moreover, a self-learning approach is proposed as an alternative to conventional data augmentation approaches and assessed in the context of multi-modal pain assessment based on bio-physiological signals.

## 3. Materials and Methods

Similarly to conventional Auto-Encoders (AEs) (Hinton and Zemel, 1994; Hinton and Salakhutdinov, 2006), a DDCAE consists of an encoder and a decoder. Both encoder and decoder are Convolutional Neural Networks (CNNs), whereby the encoder maps its input into a low dimensional latent space, while the decoder is optimized to reconstruct the encoder's input, based on the computed latent space representation. Moreover, the encoder's input consists of a corrupted signal (which is generated by adding a noisy signal to the clean and unaltered input signal). The parameters of both encoder and decoder are therefore optimized to reduce the reconstruction error between the decoder's output and the unaltered original input signal. The resulting robust bottleneck representation can be subsequently used to train a specific inference model.

### 3.1. Multi-Modal Deep Denoising Convolutional Auto-Encoder

**Figure 1:**
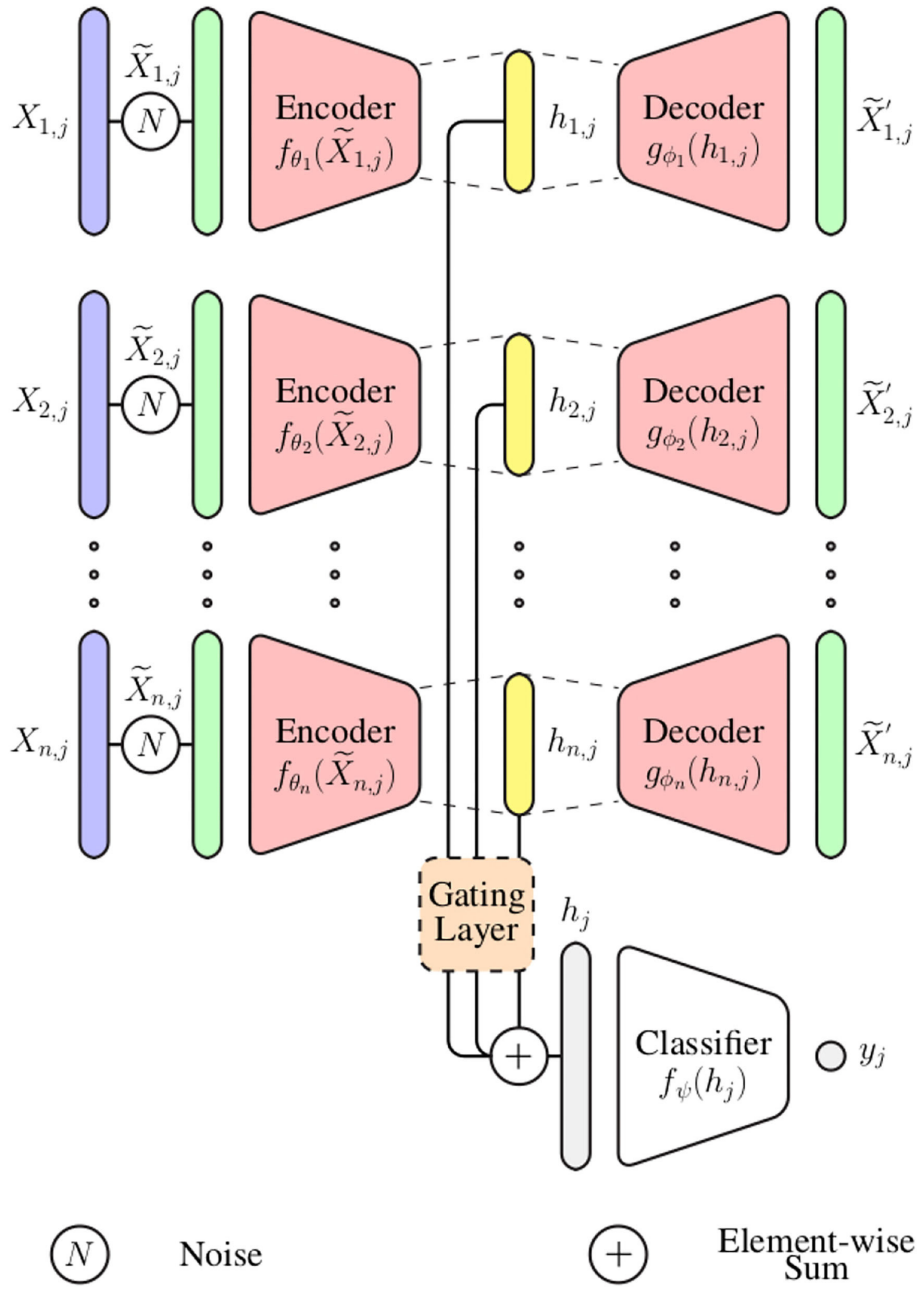
Multi-modal deep denoising convolutional auto-encoder (DDCAE) architecture.

In the current work, an information fusion architecture based on DDCAEs is proposed to perform the aggregation of information stemming from a set of diverse bio-physiological channels in the context of pain assessment. The proposed architecture, which is depicted in Figure 1, consists of learning a single latent representation for each input channel, while simultaneously optimizing a gating layer to generate a single weighted representation based on the generated channel specific latent representations. The generated weighted representation is subsequently used to optimize an inference model performing either the classification or the regression task at hand (the inference model in this case is a feed-forward neural network). The whole architecture is trained in an end-to-end manner.

In the following paragraph, the parameter *i* points at the *ith* input channel with *i* ∈ ℕ and *n* ∈ ℕ depicts the total number of input channels (1 ≤ *i* ≤ *n*). The parameter *j* points at the *jth* training sample and N∈ℕ depicts the total number of training samples (1≤j≤N). Therefore, the training set specific to the *ith* input channel can be represented as follows: ${\left\{X_{i,j}\in {\mathbb{R}}^{1\times m}\right\}}_{j=1}^{N}$ (the parameter *m* ∈ ℕ depicts the dimensionality of the training samples, since each of them consists of a 1-dimensional bio-physiological signal).

For each channel *i* ∈ ℕ, a set of noisy input signals ${\left\{{\tilde{X}}_{i,j}\in {\mathbb{R}}^{1\times m}\right\}}_{j=1}^{N}$ is first generated by altering the original signals ${\left\{X_{i,j}\in {\mathbb{R}}^{1\times m}\right\}}_{j=1}^{N}$. Each noisy signal is subsequently fed into the corresponding encoder *f*_θ_*i*__ in order to generate the latent representation *h*_*i, j*_:

(1) $$\begin{array}{l} h_{i,j}=f_{\theta_{i}}\left({\tilde{X}}_{i,j}\right) \end{array}$$

with θ_*i*_ corresponding to the set of trainable parameters of the encoder specific to the *ith* channel. The generated latent representation is further fed into the decoder *g*_ϕ_*i*__, which generates an output ${\tilde{X}}_{i,j^{\prime }}$:

(2) $$\begin{array}{l} {\tilde{X}}_{i,j^{\prime }}=g_{\phi_{i}}\left(h_{i,j}\right) \end{array}$$

**Figure 2:**
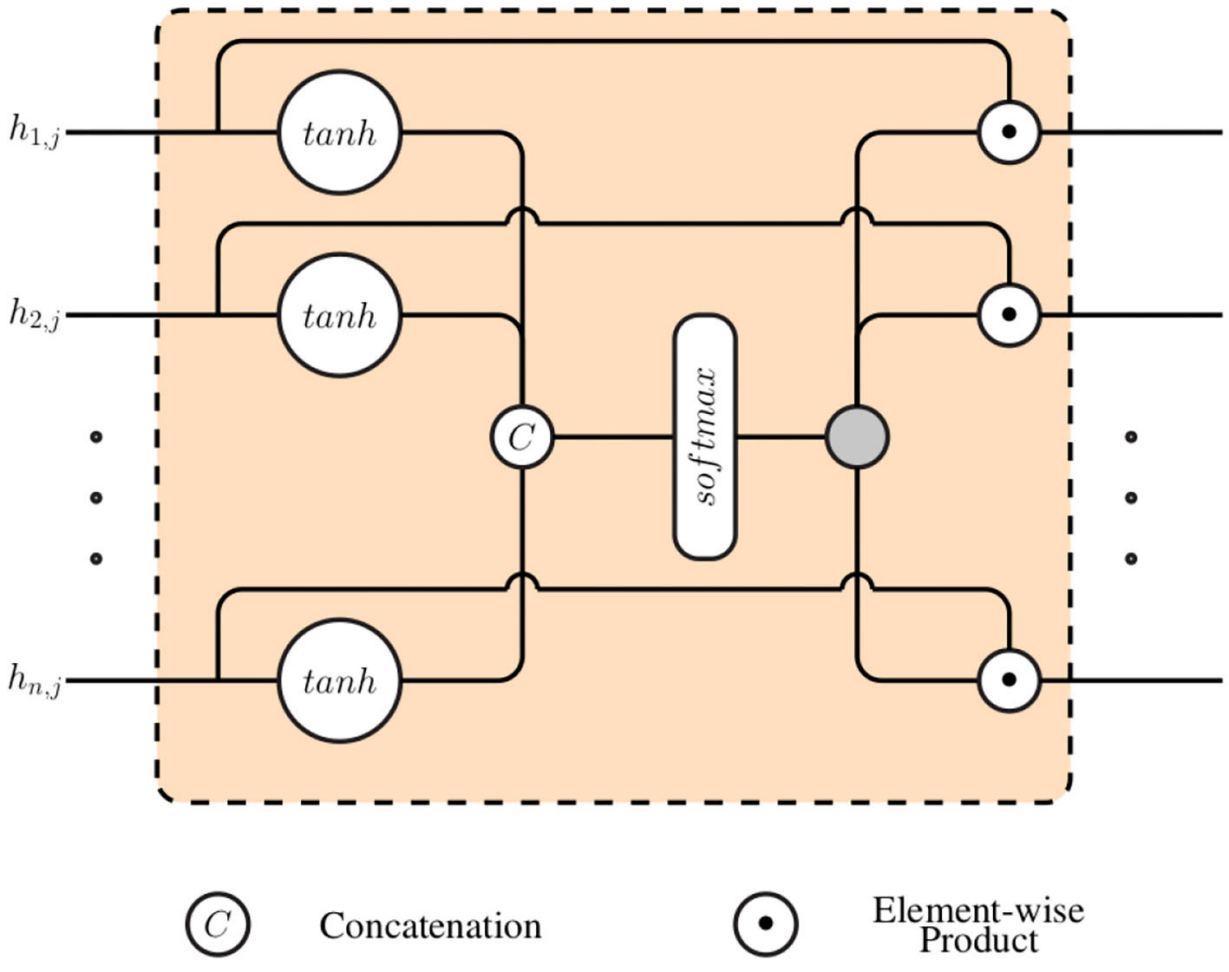
Gating layer.

Subsequently, a gating layer, which is depicted in Figure 2, is used to generate a single weighted representation based on the generated modality specific latent representations $h_{i,j}\in {\mathbb{R}}^{d_{i}}$. This approach requires that all latent representations have the same dimensionality: ∀*i* ∈ {1, 2, ⋯ , *n*}, *d*_*i*_ = η ∈ ℕ. Each latent representation *h*_*i, j*_ is first normalized by going through a layer with an hyperbolic tangent activation function (*tanh*):

(3) $$\begin{array}{l} {\hat{h}}_{i,j}=tanh\left({\hat{W}}_{i}h_{i,j}+{\hat{b}}_{i}\right) \end{array}$$

where the trainable parameters of the normalization layer consist of ${\hat{W}}_{i}\in {\mathbb{R}}^{\eta \times \eta }$ and ${\hat{b}}_{i}\in {\mathbb{R}}^{\eta }$.

The resulting normalized outputs are subsequently concatenated into a single vector $\hat{h_{j}}=\left[{\hat{h}}_{1,j},\cdots \;,{\hat{h}}_{n,j}\right]$ ($\hat{h_{j}}\in {\left[-1,1\right]}^{n\cdot \eta }$) and fed into a layer with a softmax activation function, in order to generate the weights specific to each specific feature:

(4) $$\begin{array}{l} w_{k}=\frac{exp\left(W_{k}{\hat{h}}_{j}+b_{k}\right)}{\sum_{l=1}^{n\cdot \eta }exp\left(W_{l}{\hat{h}}_{j}+b_{l}\right)} \end{array}$$

where the trainable parameters specific to the softmax layer consist of $W_{k}\in {\mathbb{R}}^{n\cdot \eta }$ and *b*_*k*_ ∈ ℝ, with 1 ≤ *k* ≤ *n* · η. The final weighted representation is subsequently generated through a weighted sum of all channel specific latent representation (*h*_*i, j*_), using the computed weights ($w={\left\{w_{k}\right\}}_{k=1}^{n\cdot \eta }$):

(5) $$\begin{array}{l} h_{j}=\overset{n}{\underset{i=1}{⊕}}\hat{w_{i}}⊙h_{i,j} \end{array}$$

where $\hat{w_{i}}\in {\mathbb{R}}^{\eta }$ and $\hat{w_{i}}=\left\{w_{\left(i-1\right)\cdot \eta +1},w_{\left(i-1\right)\cdot \eta +2},\cdots \;,w_{\left(i-1\right)\cdot \eta +\eta }\right\}$. Also, ⊙ denotes the element-wise product, while ⊕ denotes the element-wise sum. $h_{j}\in {\mathbb{R}}^{\eta }$ is the resulting weighted representation, which is further fed into an inference model *f*_Ψ_ to perform either a classification or regression task [*y*_*j*_ = *f*_Ψ_(*h*_*j*_)].

The parameters of the DDCAEs are optimized to minimize the reconstruction error between each decoder's output ${\tilde{X}}_{i,j^{\prime }}$ and the original unaltered signal *X*_*i, j*_. In the current work, the reconstruction error consists of the mean squared error function:

(6) $$\begin{array}{l} E_{i}=\frac{1}{N}\overset{N}{\underset{j=1}{\sum }}{\left\|X_{i,j}-{\tilde{X}}_{i,j^{\prime }}\right\|}_{2}^{2}+\lambda {\left\|W_{i}\right\|}_{2}^{2} \end{array}$$

where $\lambda {||W_{i}||}_{2}^{2}$ represents a regularization term, with *W*_*i*_ = {θ_*i*_, ϕ_*i*_} representing the set of all trainable parameters of the DDCAE specific to the *ith* modality. The parameters of the inference model are optimized accordingly to the task at hand. In the case of a classification task, the corresponding loss function is the categorical cross-entropy loss:

(7) $$\begin{array}{l} L_{f_{\text{Ψ}}}=-\overset{c}{\underset{cl=1}{\sum }}y_{cl}log\left(\hat{y_{cl}}\right) \end{array}$$

where *y*_*cl*_ is the ground-truth value of the *cl*^*th*^ class and $\hat{y_{cl}}$ is the corresponding classification output value (*c* ∈ ℕ corresponds to the total number of classes). In the case of a regression task, the corresponding loss function is the mean squared error function:

(8) $$\begin{array}{l} L_{f_{\text{Ψ}}}=\frac{1}{N}\overset{N}{\underset{j=1}{\sum }}{\left\|f_{\text{Ψ}}\left(h_{j}\right)-y_{j}\right\|}_{2}^{2} \end{array}$$

Since the entire architecture is trained in an end-to-end manner, the entirety of the parameters are optimized by minimizing the following objective function:

(9) $$\begin{array}{l} L=\overset{n}{\underset{i=1}{\sum }}\alpha_{i}E_{i}+\alpha_{\text{Ψ}}L_{f_{\text{Ψ}}} \end{array}$$

where the parameters α_*i*_ and α_Ψ_ are regularization weights assigned to the loss functions specific to each of the models.

### 3.2. Attention Mechanism

**Figure 3:**
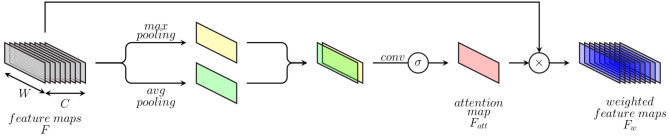
Attention mechanism.

Inspired by the dynamics involved in visual perception (Luck and Ford, 1998), artificial attention mechanisms consist of approaches designed in order to perform the assessment and selection of relevant visual cues, accordingly to the underlying visual task. An intelligent selective processing of specific regions of interest has proven to be very effective and able to significantly improve the performance of deep neural networks for visual computing tasks in areas such as visual image captioning (Chen et al., 2017), object detection (Woo et al., 2018), and image segmentation (Zhao et al., 2020). Inspired by such approaches, the previously described DDCAEs (see section 3.1) are extended with a 1-dimensional attention mechanism, in order to focus on the most relevant feature descriptors accordingly to the task at hand, therefore improving the overall performance of the inference model. The proposed attention mechanism (which is depicted in Figure 3) consists of an extension to 1-dimensional feature maps of a spatial attention module for 2-dimensional feature maps originally proposed in Woo et al. (2018). The attention mechanism aims at generating a weighted representation of the optimized feature maps (or feature representations) according to the relevance of each feature descriptor for the specific task at hand.

This is done by generating a specific weighting mask through a channel-wise aggregation of information, followed by a specific convolution operation with a sigmoid activation function, therefore highlighting the relevance of specific regions of interest within the feature maps and weighting these regions accordingly. The attention mechanism consists of first applying average-pooling and max-pooling operations over the channel axis of a set of feature maps stemming from an intermediate convolution layer. The resulting feature maps from both pooling operations are subsequently concatenated and fed into a convolution layer with a sigmoid activation function in order to generate an attention map (see Equation 10). Finally, weighted feature maps are generated by performing an element-wise multiplication of the initial feature maps with the computed attention map (see Equation 11).

More specifically, given a set of feature maps *F* ∈ ℝ^1 × *W* × *C*^ (where *W* ∈ ℕ_>0_ depicts the length of the feature maps and *C* ∈ ℕ_>0_ depicts the number of feature maps) stemming from an intermediate convolution layer, an attention feature map $F_{att}\in {\mathbb{R}}^{1\times W}$ is generated by using both max-pooling and average-pooling operations applied across the channels of the set of feature maps as follows:

(10) $$\begin{array}{l} F_{att}=\sigma \left(f^{1\times kernel\;size}\left(\left[AvgPool\left(F\right),MaxPool\left(F\right)\right]\right)\right) \end{array}$$

where *f*^1×*kernelsize*^ depicts a 1-dimensional convolution operation with a filter size of 1 × *kernel* *size* (which is applied on a concatenation of the feature maps resulting from both average- and max-pooling operations) and σ depicts the sigmoid function. The weighted feature maps $F_{w}\in {\mathbb{R}}^{1\times W\times C}$ are subsequently generated as follows:

(11) $$\begin{array}{l} F_{w}=F⊗F_{att} \end{array}$$

where ⊗ depicts an element-wise multiplication. During this operation the values of the attention map are broadcasted along the channels of the feature maps. This specific attention mechanism is integrated into the previously described multi-modal DDCAE architecture (see Figure 1) and is applied following each convolution layer in the modality specific encoder architectures.

### 3.3. Self-Supervised Learning of Physiological Signals

In the well-known supervised learning setting, the learner faces a data set $D={\left\{\left(x_{i},y_{i}\right)\right\}}_{i=1}^{N}$ consisting of *N* pairs of training data *x* and corresponding labels *y* and must find a hypothesis that minimizes some loss on the dataset. Collecting labels is expensive and time consuming, especially in the context of pain assessment, as additional experiments have to be conducted. To remedy this, relaxations of supervised learning have been proposed, such as semi-supervised learning (Schwenker and Trentin, 2014), where we only have label information for a subset of data points, and unsupervised learning (Barlow, 1989), where no labels are available. A new approach is Self-Supervised Learning (Jing and Tian, 2020; Jaiswal et al., 2021), which is a form of representation learning (Bengio et al., 2013). Such Self-Supervised Learning algorithms usually consist of a pretext task, that we use to learn a data representation and a downstream task that is the final supervised learning task.

In this study, we follow this line of work and propose an information-theoretic approach to learn such an informative representation. To this end, we propose three additions to the architecture introduced so far. Firstly, we introduce Adaptive Variational Auto-Encoders (AVAE) for self-supervised learning, that *learn* a data dependent prior over their latent representation as opposed to using a fixed prior (Kingma and Welling, 2014). We argue, that this information-processing bottleneck enforces an optimal trade-off between representational capacity and information-processing cost as measured by the Kullback-Leibler divergence (D_KL_) between the latent posterior *p*(*h*|*x*) and its prior *p*(*h*). This idea has been introduced by Hihn et al. (2018) and has shown promising results on low dimensional data. Secondly, we propose to use information processing constraints on the gating layer and the classifier based on a theory of bounded rationality (Ortega et al., 2015). To this end, we follow the work of Hihn and Braun (2020b), where they introduce and motivate such constraints and show their favorable effects on generalization in the meta-learning setting (Hihn and Braun, 2020a). We show that these types of constraints enable efficient representation learning, which is the pretext task. Lastly, we propose to use the learned representation to generate artificial data and use this data to fine-tune the model, which is the downstream task.

#### 3.3.1. Adaptive Variational Auto-Encoders

In the following sections we introduce the Adaptive Variational Auto-Encoder and its self-supervised learning application, in order to improve automatic pain assessment while requiring less data points then conventional data augmentation techniques.

Variational Auto-Encoders (VAE) (Kingma and Welling, 2014) are generative models that build on deterministic Auto-Encoder networks. They are best understood as variational Bayesian inference in a latent variable model *p*(*x*|*h*) with a prior distribution *p*(*h*), where *x* represents the observable data, and *h* the latent variable that explains the data. The goal is to find a set of parameters φ^*^ that maximize the data likelihood *p*_φ_(*x*) = ∫*p*_φ_(*x*|*h*)*p*(*h*)d*h*. We can draw samples from *p*_φ_(*x*) by first sampling *h* and then draw *x* from *p*_φ_(*x*|*h*). As maximum likelihood optimization is intractable due to the integral, we express the likelihood in a different form by defining a variational distribution *q*(*h*|*x*) [also known as Evidence Lower Bound (ELBO)], such that

(12) $$\begin{array}{l} \log p_{\varphi }\left(x\right)\geq \int q\left(h|x\right)\log\frac{p_{\varphi }\left(x|h\right)p\left(h\right)}{q\left(h|x\right)}\text{d}h=:\text{F}\left(\varphi \right). \end{array}$$

Assuming *q*(*h*|*x*) is expressive enough to approximate the true posterior distribution *p*_φ_(*h*|*x*) well, we can directly maximize the lower bound F(φ) by gradient descent. In VAEs, *q*(*h*|*x*) is the encoder network that generates a latent representation *h* given and input *x*, and *p*(*x*|*h*) is the decoder that reconstructs *x* from *h*. We assume all distributions to be isotropic Gaussians.

We extend this approach by removing the fixed prior *p*(*h*) and allow for an adaptive prior. To learn a prior that allows for efficient information processing by minimizing the D_KL_ between *p*_φ_(*h*|*x*) and *p*(*h*), we define *p*(*h*) to be the marginal of *p*_φ_(*h*|*x*) over the inputs *x*:

(13) $$\begin{array}{l} p\left(h\right)=\int_{x\in X}p_{\varphi }\left(x|h\right)\text{d}x. \end{array}$$

This term is not tractable as the data generating distribution *p*(*x*) is unknown. We approximate the true marginal by running an exponential running mean with window length τ (Hihn and Braun, 2020b; Leibfried and Grau-Moya, 2020):

(14) $$\begin{array}{l} q_{t+1}\left(h\right)=\left(1-\frac{1}{\tau }\right)q_{t}\left(h\right)+\frac{1}{\tau }p_{\varphi }\left(h|x_{t}\right), \end{array}$$

where *q*_*t*_(*h*) is the approximated marginal after observing *t* samples. To find the optimal parameters φ^*^, we optimize the following variational Auto-Encoder objective:

(15) $$\begin{array}{r} \varphi^{*}=\underset{\varphi }{\text{arg max}}{\;𝔼}_{x∽p\left(x\right),h∽p_{\varphi }\left(h|x\right)}\left[\log p_{\varphi }\left(x|h\right)\right]\\ -\frac{1}{\beta_{1}}{\text{D}}_{\text{KL}}\left[p_{\varphi }\left(h|x\right)\|q\left(h\right)\right], \end{array}$$

where β_1_ governs a trade-off between maximizing the log-likelihood and keeping the variational posterior close to the prior *q*(*h*). We will refer to *q*(*h*) as *p*(*h*) to keep notation consistent. There are different interpretations of this approach, e.g., learning with information constraints (Hihn et al., 2018, 2019; Hihn and Braun, 2020b), meta-learning (Hihn and Braun, 2020a), tempered posteriors in variational Bayes applications (Aitchison, 2021), and learning disentangled representations (Higgins et al., 2016). The additional degree of adaptivity introduced allows to learn data dependent priors which can improve the quality of generated samples, as we will show empirically in section 4.3.

#### 3.3.2. Representation Learning

**Figure 4:**
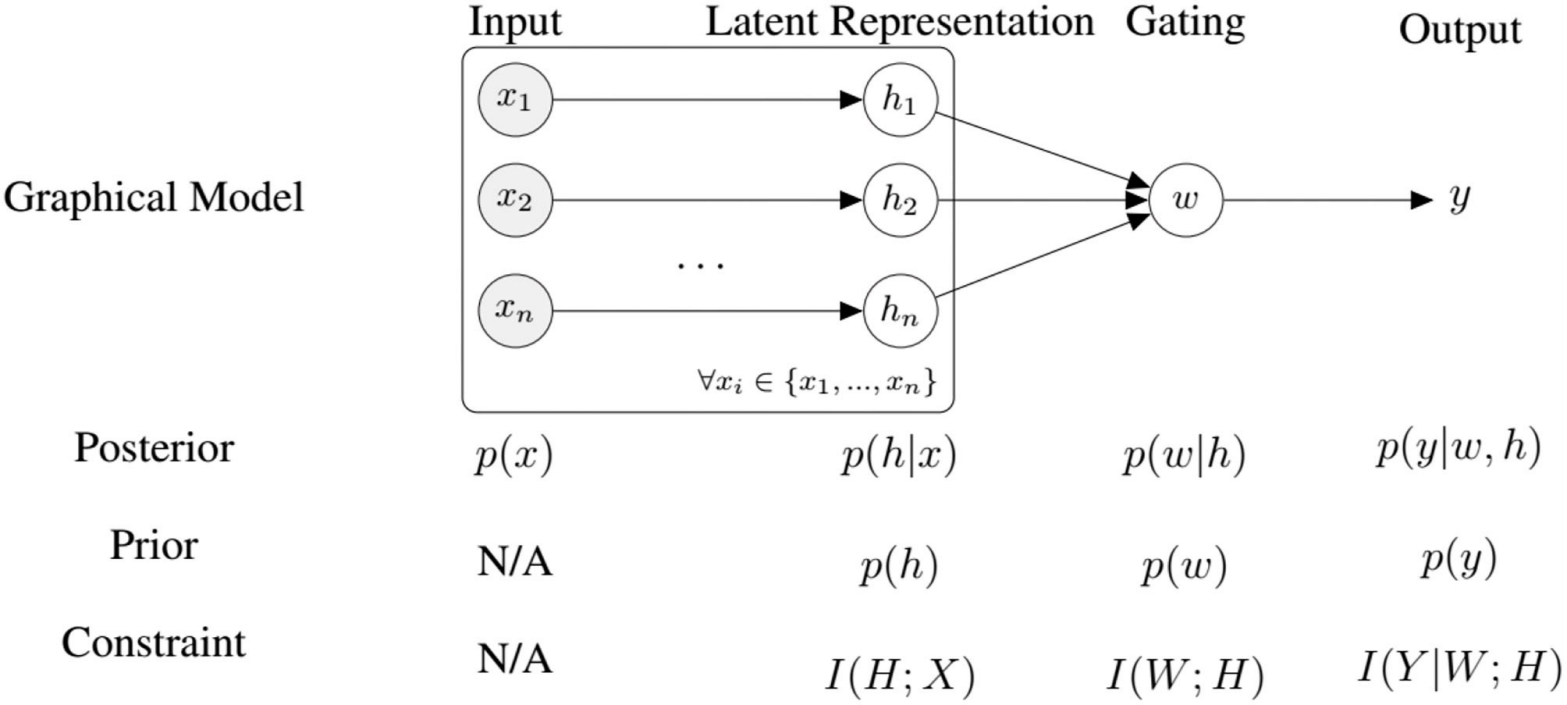
An overview of our model for self-supervised fine-tuning and the corresponding information-processing constraints, where *n* is the number of modalities.

We can interpret the architecture introduced in section 3.1 as an encoder-decoder structure thats maps a high dimensional input signal *x* to a low dimensional latent representation *h*. Classification is then performed only using a non-linear combination of the low dimensional representation. Training this architecture in an end-to-end fashion will produce values of *h* that both minimize the reconstruction error (in other words, capture the data well) and the classification error. To further improve this coupling, we propose to impose information-processing constraints on the latent representation, the gating layer, and on the classifier. We argue that such constraints encourage the system to learn representations that discover regularities in the data by discarding all unnecessary information (Hihn and Braun, 2020b). To this end, we formulate an information-theoretic coupling as a two stage hierarchical system (Genewein et al., 2015) with the following objective function:

(16) $$\begin{array}{l} \underset{p\left(w|h\right),p\left(y|w,h\right)}{\max}𝔼\left[L\left(x,y\right)\right]-\frac{1}{\beta_{2}}I\left(W;H\right)-\frac{1}{\beta_{3}}I\left(Y|W;H\right), \end{array}$$

where L(x,y) is a loss function, *x* ∈ *X* the input, *y* ∈ *Y* the output, *h* ∈ *H* the latent representations produced by the Auto-Encoders, *W* the weights of the gating layer (see Equation 4), and *I*(*X*; *Y*) is the mutual information between random variables *X* and *Y*. The hyper-parameters β_2_ and β_3_ are Lagrange multipliers that govern the trade-off between information-processing cost and utility as measured by the loss L. Note that the classifier output *y* depends on the combined latent variables *h*, as described by Equation (5). We can rewrite Equation (16) into

(17) $$\begin{array}{l} \underset{\theta ,\vartheta }{\max}𝔼\left[L\left(x,y\right)-\frac{1}{\beta_{2}}\log\frac{p_{\theta }\left(w|h\right)}{p\left(w\right)}-\frac{1}{\beta_{3}}\log\frac{p_{\vartheta }\left(y|w,h\right)}{p\left(y\right)}\right], \end{array}$$

where *h* is the latent representation, *x* the input, *y* the output, *p*(*w*|*h*) is some fusion policy and *p*(*y*|*w, h*) is the output of a decision-maker (e.g., a classifier), and θ, ϑ are the parameters. This formulation allows us to perform updates in an on-line manner. As outlined earlier, the optimal priors to find an optimal trade-off are the marginals of the posterior policies *p*(*w*|*h*) and *p*(*y*|*w, h*), which we approximate by a running mean average. Combining VAE and representation learning losses we have the following objective function allowing us to train the system end-to-end (see Figure 4):

(18) $$\begin{array}{r} \underset{\varphi ,\theta ,\vartheta }{\max}𝔼[L\left(x,y\right)-\log p_{\varphi }\left(x|h\right)-\frac{1}{\beta_{1}}\log\frac{p_{\varphi }\left(h|x\right)}{p\left(h\right)}\\ -\frac{1}{\beta_{2}}\log\frac{p_{\theta }\left(w|h\right)}{p\left(w\right)}-\frac{1}{\beta_{3}}\log\frac{p_{\vartheta }\left(y|w,h\right)}{p\left(y\right)}]. \end{array}$$

#### 3.3.3. Self-Supervised Fine Tuning

The method we propose aims to find a representation that allows us to generate informative data samples, without having to go through an expensive data generation and labeling process. The self-supervised fine tuning algorithm we propose consists of a training phase (called the pretext task), followed by a generative and fine tuning phase (also called the downstream task). Firstly, we train the whole system, namely an adaptive variational Auto-Encoder for each modality and the classifier (see Figure 1) using the full (but not augmented) dataset (see Tables 5, 6 for an account of samples used and generated). The main goal of this phase is for the generative models to learn a latent representation *h* per modality that is beneficial to the classification task that uses a combination of the latent representations of the Auto-Encoders, i.e., *p*(*y*|*w, h*), where *y* is the output label, *h* represents the latent variables, and *w* represents the weights computed by the gating layer. In this way the learned posterior *p*(*h*|*x*), where *h* is the latent variable and *x* the input signal, optimizes both the signal reconstruction architecture and the classification model simultaneously, by optimizing the Auto-Encoder's objective function given by Equation (15). The resulting representation thus captures the structure of the data, as well as the semantic information, making it a suitable candidate for a data generation process. We give an overview of our technique in Algorithm 1.

**Algorithm 1:** We split Self-Supervised Learning into three phases: (i) training, (ii) self-supervised fine-tuning, and (ii) evaluation. $D_{-j}$ denotes the dataset without data from subject *j*.

1: **Input**: Dataset D with data from *N*_*s*_ subjects with *L* modalities
2: **Hyper-parameters**: prior penalty parameters β_1_, β_2_, β_3_, number of samples *K* to generate, training episodes *N*, fine-tuning training episodes *M*
3: **for** *j* = 1, 2,., *N*_*s*_ **do**
4: Initialize Auto-Encoders and classifier parameters
5: Train Auto-Encoders and classifier for *N* episodes using subset $D_{-j}$ with parameters β_1_, β_2_, β_3_
6: $D_{\text{self-super}}=\emptyset$
7: **for** *i* = 1, 2,., *K* **do**
8: **for** *l* = 1, 2, ..., *L* **do**
9: Generate latent representation *h*_*l*_ using prior *p*_*l*_(*h*)
10: Reconstruct samples ${\tilde{X}}_{l}$ by using the decoders *g*_ϕ_*l*__(*h*)
11: **end for**
12: Combine all ${\tilde{X}}_{l}$ into ${\tilde{X}}_{i}$
13: Classify sample using *f*_φ_(**h**), where **h** is the output of the gating layer, to obtain label ỹ_*i*_
14: $D_{\text{self-super}}=D_{\text{self-super}}\cup \left({\tilde{X}}_{i},{ỹ}_{i}\right)$
15: **end for**
16: Fine-tune system using only $D_{\text{self-super}}$ for *M* episodes with parameters β_1_, β_2_, β_3_
17: Evaluate data from subject *j* and collect metrics
18: **end for**
19: **return** evaluation metrics

### 3.4. Data Sets

**Figure 5:**
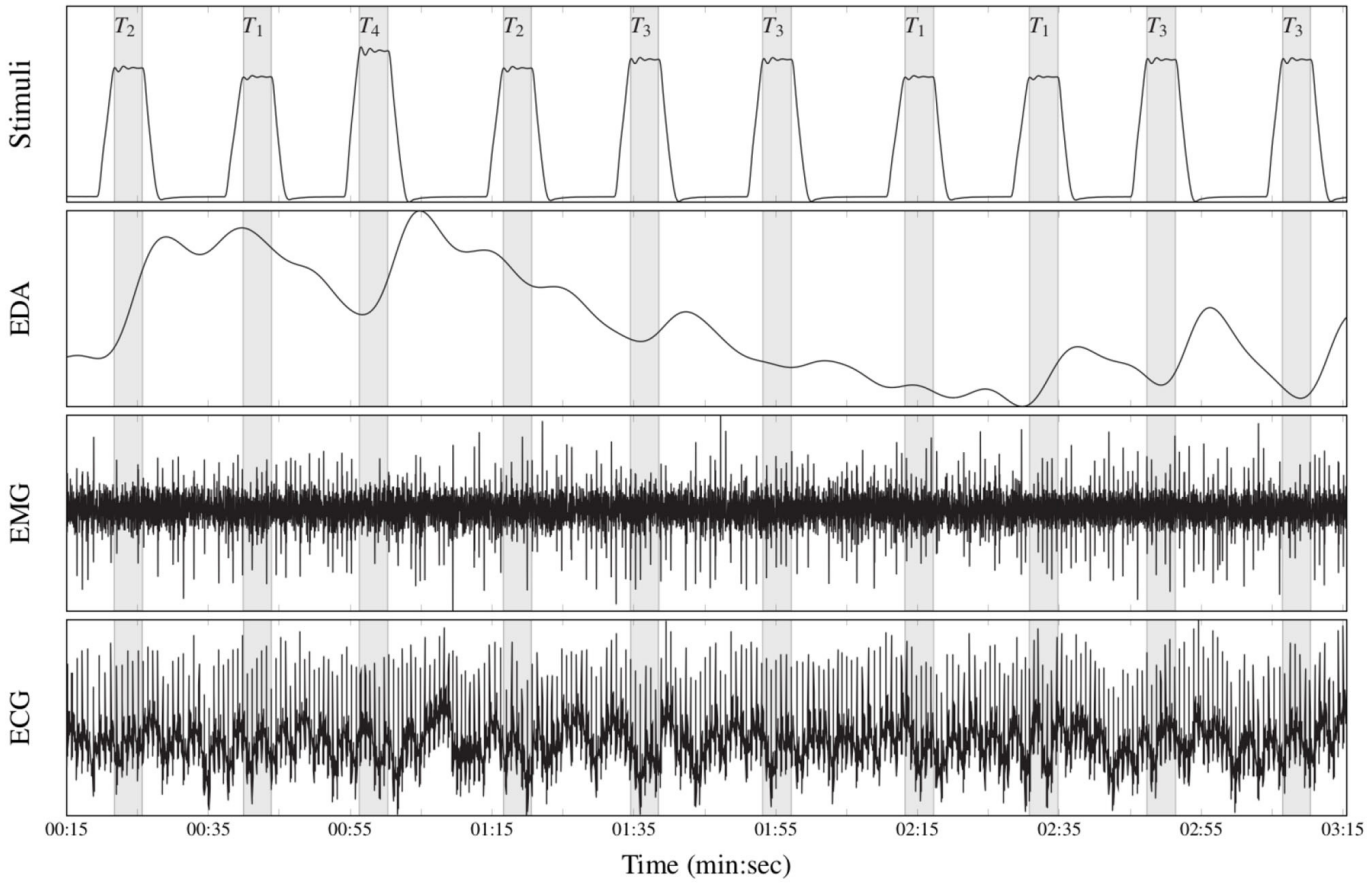
Recorded physiological data (BioVid Heat Pain Database, thermal pain elicitation). From top to bottom: stimuli (*T*_1_: pain threshold temperature, *T*_2_: first intermediate elicitation temperature, *T*_3_: second intermediate elicitation temperature, *T*_4_: pain tolerance temperature); EDA (μS); EMG (μV); ECG (μV).

The BioVid Heat Pain Database (Part A) (Walter et al., 2013) is a multi-modal data set consisting of 87 healthy participants subjected to four levels of gradually increasing and individually calibrated thermal pain elicitation (*T*_1_, *T*_2_, *T*_3_, *T*_4_). Several modalities were recorded during the experiments including video streams, EDA, ECG, and EMG signals. Each single level of pain elicitation was randomly elicited a total of 20 times, with each elicitation lasting 4 s (sec), followed by a recovery phase of randomized duration (lasting between 8 and 12 s). During this recovery phase, a baseline temperature *T*_0_ of 32°C was applied (see Figure 5). The data set specific to each participant consists of a total of 20 × 5 = 100 samples, summing up to a database of 87 × 100 = 8, 700 samples. Each sample is labeled with its corresponding level of thermal pain elicitation (*T*_0_, *T*_1_, *T*_2_, *T*_3_, *T*_4_). The proposed approaches are evaluated uniquely on the physiological signals EMG, ECG, and EDA.

Analogously to the BioVid Heat Pain Database, the SenseEmotion Database (Velana et al., 2017) consists of 45 healthy individuals subjected to 3 levels of individually calibrated and gradually increasing thermal pain elicitation (*T*_1_, *T*_2_, *T*_3_) and a baseline level *T*_0_ set identically for all participants to 32°C (corresponding to no pain elicitation). The modalities recorded during the performed experiments consist of audio signals, 3 video streams, the trapezius EMG signal, RSP, ECG, and EDA signals. The performed experiments consist of two 40 min sessions, during which the piece of hardware used to perform the thermal pain elicitations was attached to a specific forearm (once on the right forearm and once on the left forearm). The calibration of the temperatures of elicitation as well as the thermal elicitation procedure were carried out identically to the BioVid Heat Pain Database, with the only difference being the total number of stimuli per pain level. Each pain level was randomly elicited a total of 30 times with a pause of about 8–12 s between the elicitations. Due to technical issues during the experiments 5 participants were excluded from the data set because of missing or erroneous data. We therefore evaluate the proposed approaches on a reduced subset consisting of 40 participants and a data set consisting of a total of 40 × 30 × 4 × 2 ≈ 9, 600 samples. The assessment of the proposed approaches is performed uniquely on the physiological signals EMG, ECG, EDA, and RSP.

### 3.5. Data Preprocessing

Similar preprocessing operations were applied on the recorded physiological signals of both datasets. First of all, the sampling rate of the recorded signals was reduced to 256 Hz in order to significantly reduce the amount of computational requirements. Next, the amount of noise and artifacts within each signal was reduced by applying specific signal processing techniques. For both datasets, a low-pass Butterworth filter of order 3 with a cut-off frequency of 0.2 Hz was applied on the EDA signals. Concerning the BioVid Heat Pain Database, EMG signals were filtered using a fourth order bandpass Butterworth filter with a frequency range of [20, 250] Hz, while ECG signals were filtered with a third order bandpass Butterworth filter with a frequency range of [0.1, 250] Hz. Subsequently, piecewise detrending of the filtered ECG signals was performed, by subtracting a fifth degree polynomial least-squares fit from the filtered signals (as proposed in Thiam et al., 2019a). Concerning the SenseEmotion Database, the RSP signals were smoothed using a third order low-pass Butterworth filter with a cut-off frequency of 0.8 Hz. Both EMG and ECG signals were preprocessed by applying a third order bandpass Butterworth filter with respective frequency ranges of [0.05, 25] and [0.1, 25] Hz, followed by a similar piecewise detrending as in the case of the BioVid Heat Pain Database. The resulting filtered signals were subsequently segmented, and each segment in combination with its corresponding level of pain elicitation was used to perform the assessment of the proposed approaches.

**Figure 6:**
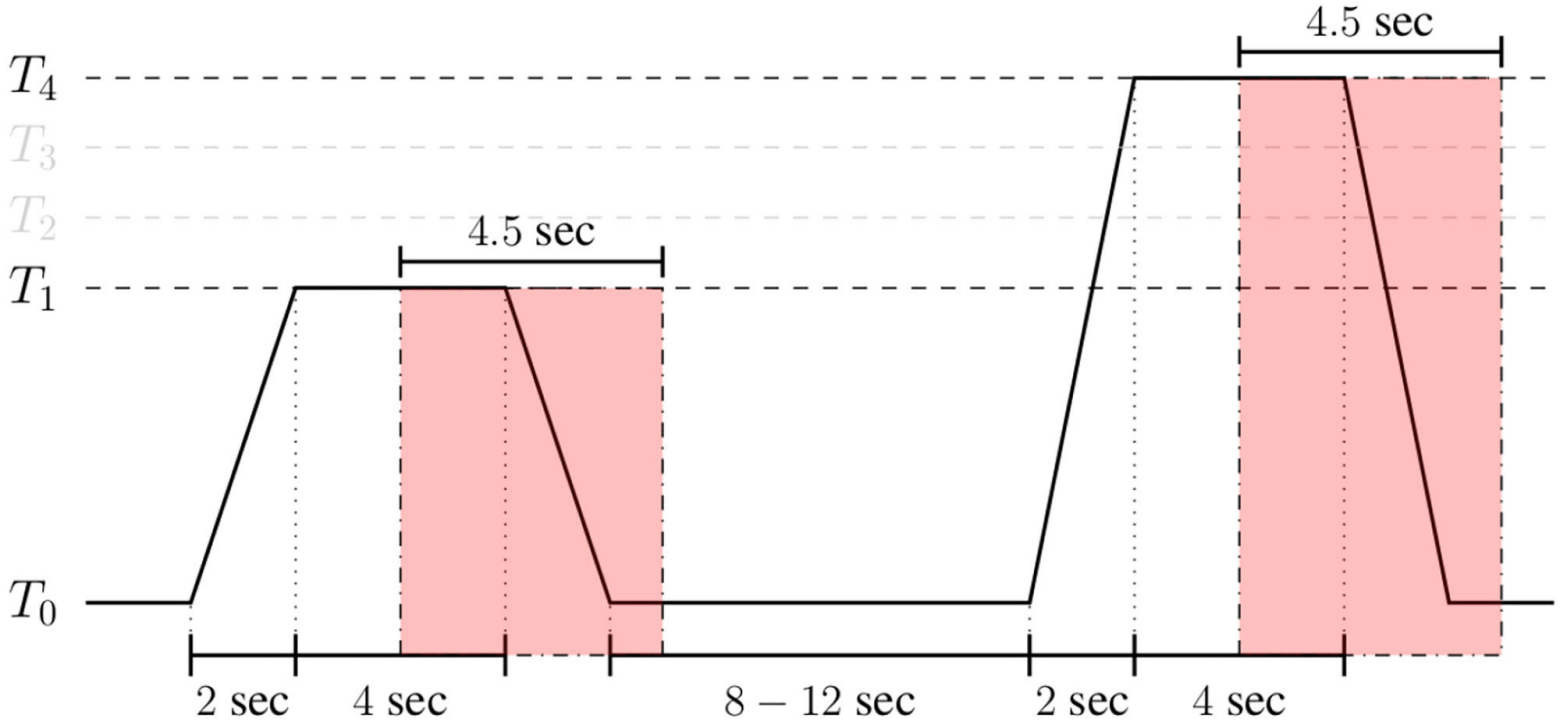
Signal segmentation (BioVid Heat Pain Database). Experiments are carried out on windows of length 4.5 s with a temporal shift of 4 s from the elicitations' onset.

In the case of the BioVid Heat Pain Database, the assessment is performed on windows of length 4.5 s with a shift of 4 s from the elicitations' onset (see Figure 6). Analogously, in the case of the SenseEmotion Database, the assessment of the proposed approaches is performed on widows of length 6.5 s with the same shift of 4 s from the elicitations' onset. Each signal within these specific windows consists of a one-dimensional array of size *m* = 4.5 × 256 = 1, 152 for the BioVid Heat Pain Database, and *m* = 6.5 × 256 = 1, 664 for the SenseEmotion Database. Moreover, since a huge amount of parameters specific to the multi-modal DDCAE architectures has to be optimized, data augmentation was performed by shifting the 4.5 s (6.5 s, respectively) window of segmentation backward and forward in time with small shifts of 250 ms and a maximum total window shift of 1 s in each direction. These shifts were performed, starting from the initial position of the windows (as depicted in Figure 6). This procedure was performed uniquely during the training phase of the proposed architectures, resulting in generating nine times the total amount of training samples specific to the initial windows of segmentation. Following the optimization of the multi-modal DDCAE architectures, the evaluation of the trained architectures was performed on the initial windows of 4.5 s (6.5 s, respectively) with a shift of 4 s from the elicitations' onset.

## 4. Results

In the following section, a description of the results relative to the proposed multi-modal DDCAE architecture is provided. An assessment of the performance of the architecture is performed and a comparison of the results achieved with and also without the proposed attention mechanism is conducted. Finally, the results specific to the proposed self-learning algorithm as well as a comparison of the achieved results between self-learning approaches and fully supervised learning approaches are described and discussed.

### 4.1. Experimental Settings

In the current work, the multi-model DDCAE architecture consists of one-dimensional convolutional operations. The Exponential Linear Unit (ELU) (Clevert et al., 2016) function defined in Equation (19) (with α = 1) is used in both convolutional and fully connected layers as activation function, except for the output layer of both classification and regression models. In the case of a classification model, a softmax activation function is used, while a linear activation function is used in the case of a regression model.

(19) $$\begin{array}{l} elu_{\alpha }\;(x)=\{\begin{array}{ll} \alpha \;(exp\;(x)-1), & \text{if }x<0\\ x, & \text{if }x\geq 0 \end{array} \end{array}$$

Similar encoders' and decoders' architectures are used for each physiological signal, with the only difference being the size of the convolutional kernel for each modality specific DDCAE: in the case of EDA, a fixed convolutional kernel with a size of 3 and a stride of 1 is set empirically. In the case of EMG, ECG and RSP, the size of the convolutional kernel is set to 11 with a stride set also to 1. The dimensionality of the resulting latent representation specific to each modality specific DDCAE is set empirically to η = 256. The designed architectures are summarized in Table 1. The convolutional kernel of the attention mechanism (see Equation 10) is set empirically to 3 with a stride of 1 (*kernelsize* = 3).

**Table 1:** DDCAE architecture: the MaxPooling and UpSampling operations are performed with an identical pooling size set to 2 and a stride set to 2.

**Encoder**	
**Layer**	**No. kernels/Units**
2 × Conv1D and MaxPooling	8
2 × Conv1D and MaxPooling	16
2 × Conv1D and MaxPooling	32
Flatten	−
Fully connected	256
**Decoder**	
**Layer**	**No. kernels/Units**
Fully connected	576
Reshape	−
2 × Conv1D and UpSampling	32
2 × Conv1D and UpSampling	16
2 × Conv1D and UpSampling	8
1 × Conv1D	1
**Inference (classification or regression)**	
**Layer**	**No. kernels/Units**
Fully connected	128
Dropout	−
Fully connected	*c*

*The Dropout rate is set empirically to 0.25. Concerning the underlying inference task, in case of a classification task c depicts the number of classes, while in the case of a regression task, c = 1*.

All architectures are trained using the Adaptive Moment Estimation (Adam) (Kingma and Ba, 2015) optimization algorithm with a fixed learning rate set empirically to 10^−5^. The training process is performed through a total of 100 epoches. The batch size is set to 40 in the case of a 2*Classes* classification task and 100 in the case of a 5*Classes* classification task, for the BioVid Heat Pain Database. Concerning the SenseEmotion Database, the batch size is set to 120 for 2*Classes* classification tasks, and 480 for 4*Classes* classification tasks. The same batch sizes are used for regression tasks. The regularization parameter in Equation (6) is set as follows: λ = 10^−3^. The regularization weights of the objective function defined in Equation (9) are set as follows: α_1_ = α_2_ = α_3_ = 0.2 and α_Ψ_ = 0.4, for the BioVid Heat Pain Database; α_1_ = α_2_ = α_3_ = α_4_ = 0.15 and α_Ψ_ = 0.4, for the SenseEmotion Database. The regularization weight specific to the inference model is set higher than the others in order to focus more on the inference performance of the whole architecture. Moreover, noisy signals are generated by adding some Gaussian noise to the unaltered original signals. The parameters of the distribution specific to the Gaussian noise consist of a standard deviation set to 0.1 and a mean set to 0. The implementation and the evaluation of the proposed approaches were performed with the libraries Tensorflow (Abadi et al., 2016), Keras (Chollet et al., 2015), and Scikit-learn (Pedregosa et al., 2011). The evaluation of the approaches was performed by applying a *Leave One Subject Out* (LOSO) cross-validation evaluation, which means that the data specific to each single participant is used once to evaluate the performance of the trained model and is never seen during the training process, while the data specific to the remaining participants is used to optimize or train the model. This results in a total of 87 experiments in the case of the BioVid Heat Pain Database, and 40 experiments in the case of the SenseEmotion Database. The results specific to each inference task that are depicted in the following sections are therefore averaged across the totality of the performed experiments.

### 4.2. Multi-Modal Deep Denoising Convolutional Auto-Encoder: Results

In the current section, an assessment of the classification performance of the proposed multi-modal DDCAE approach is performed and described. The assessment consists of performing several binary and multi-class classification tasks based on both BioVid Heat Pain Database and SenseEmotion Database. A comparison of the achieved performances of the multi-modal DDCAE approach, respectively without (w/o) and with the proposed attention mechanism, is also provided. The performance measures used to conduct the assessment are described in Table 2.

**Table 2:** Classification performance measures.

**Measure**	**Binary classification**	**Multi-class classification**
Accuracy	$\frac{tp+tn}{tp+tn+fp+fn}$	$\frac{1}{c}\sum_{i=1}^{c}\frac{tp_{i}+tn_{i}}{tp_{i}+tn_{i}+fp_{i}+fn_{i}}$
Precision	$\frac{tp}{tp+fp}$	$\frac{1}{c}\sum_{i=1}^{c}\frac{tp_{i}}{tp_{i}+fp_{i}}$
Recall	$\frac{tp}{tp+fn}$	$\frac{1}{c}\sum_{i=1}^{c}\frac{tp_{i}}{tp_{i}+fn_{i}}$
F1-Score	$\frac{2\times \text{Precision}\times \text{Recall}}{\text{Precision}+\text{Recall}}$	

*In the case of multi-class classification experiments: tp_i_ corresponds to true positives, tn_i_ corresponds to true negatives, fp_i_ corresponds to false positives and fn_i_ corresponds to false negatives in the confusion matrix associated with the ith class. Since the data sets used to perform the evaluation of the proposed approaches are balanced, the macro-averaged F1-score is used in the case of multi-class classification*.

In the case of a binary classification task (e.g., *T*_0_*vs*.*T*_4_), *truepositives* (*tp*) correspond to the number of correctly classified samples of the positive class (e.g., *T*_4_), while true negatives corresponds to the number of correctly classified samples of the negative class (e.g., *T*_0_). Analogously, *falsepositives* (*fp*) correspond to the number of incorrectly classified samples of the negative class, while *falsenegatives* (*fn*) correspond to the number of incorrectly classified samples of the positive class. These values stem from the confusion matrix of an evaluated classification model and are used to define and compute the performance measures.

First of all, a summary of the results specific to the signal reconstruction performance of the proposed approach in terms of Mean Squared Error (MSE) averaged across the performed LOSO cross-validation evaluation ($\forall i,{\text{MSE}}_{i}=\frac{1}{T}\sum_{j=1}^{T}{\left\|X_{i,j}-{\tilde{X}}_{i,j}^{{}^{\prime }}\right\|}_{2}^{2}$, with $T\in {\mathbb{N}}_{>0}$ being the size of the testing set specific to the *i*^*th*^ modality) is provided in Table 3. Concerning the BioVid Heat Pain Database, the attention mechanism improves the performance of the DDCAE architectures and in most cases significantly, with regards to both EDA and ECG signals. Concerning the EMG signal, both DDCAE architectures (without and with attention mechanism) perform similarly, with the approach without attention mechanism slightly outperforming the one with the attention mechanism, however not significantly. Concerning the SenseEmotion Database, the DDCAE architectures with the attention mechanism outperform those without attention mechanism in most cases, with regards to the EDA, ECG, and EMG signals. The reconstruction error of the RSP signal is significantly higher than those of the other signals, regardless of the applied approach. A similar reconstruction error performance between both approaches with and without attention mechanism can also be seen across all classification tasks. Overall, the proposed attention mechanism has a positive effect on the reconstruction performance of the multi-modal DDCAE architecture, and helps further reducing the MSE between the output of the model and the original unaltered input signals.

**Table 3:** Signal reconstruction performance [Mean Squared Error (MSE)] comparison in a *Leave One Subject Out* (LOSO) cross-validation evaluation setting: Average MSE in % (standard deviation in %).

**BioVid Heat Pain Database (Part A)**						
Task	*T*_0_*vs*.*T*_4_		*T*_1_*vs*.*T*_4_		*T*_0_*vs*.*T*_1_*vs*.*T*_2_*vs*.*T*_3_*vs*.*T*_4_ (5Classes)	
Model	DDCAE		DDCAE		DDCAE	
	W/o attention	With attention	W/o attention	With attention	W/o attention	With attention
EDA	04.82(05.26)	03.61(03.90)^*^	05.13(05.47)	03.79(04.46)^*^	03.18(04.14)	**03.14(04.14)**
ECG	09.23(07.12)	08.57(07.32)^*^	09.31(07.54)	07.75(06.92)^*^	06.25(05.81)	05.92(05.72)^*^
EMG	**17.38(32.39)**	17.40(32.65)	17.37(31.80)	**17.13(31.63)**	**16.28(31.34)**	16.89(31.50)
**SenseEmotion Database**						
Task	*T*_0_*vs*.*T*_3_		*T*_1_*vs*.*T*_3_		*T*_0_*vs*.*T*_1_*vs*.*T*_2_*vs*.*T*_3_ (4Classes)	
Model	DDCAE		DDCAE		DDCAE	
	W/o attention	With attention	W/o attention	With attention	W/o attention	With attention
EDA	03.75(04.32)	**03.46(03.88)**	04.03(04.73)	**03.86(04.50)**	03.81(04.37)	**03.77(03.85)**
ECG	05.83(02.91)	05.52(03.13)^*^	05.74(03.09)	05.43(03.32)^*^	**05.61(03.23)**	05.54(03.23)
EMG	**07.42(09.05)**	07.62(09.41)	07.32(08.32)	07.04(08.72)^*^	07.53(09.31)	**07.77(08.40)**
RSP	34.71(89.97)	**33.63(87.89)**	34.37(82.40)^*^	35.11(86.38)	**35.39(94.68)**	35.97(97.17)

The best achieved performance is depicted in bold. An asterisk (*) depicts a significant performance improvement. The significance test is performed using a two-sided Wilcoxon-Signed-Rank test with a significance level of 5%.

**Figure 7:**
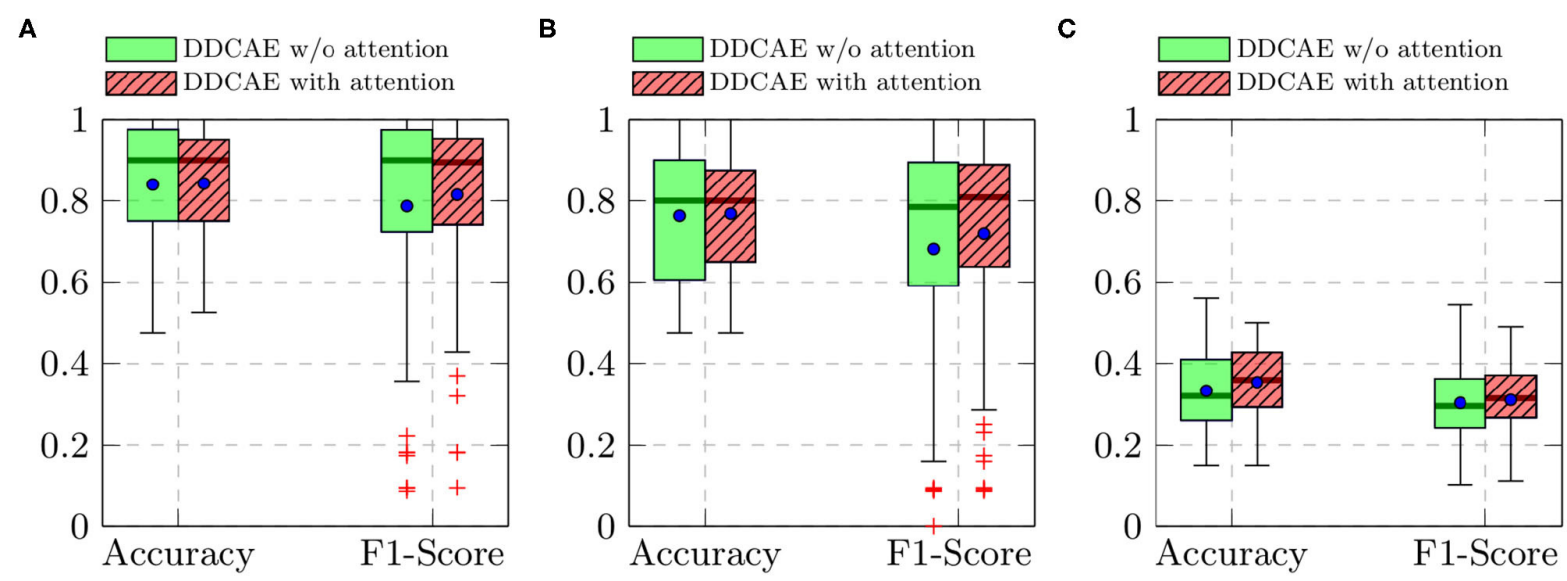
BioVid Heat Pain Database (Part A): classification performance comparison in a *Leave One Subject Out* (LOSO) cross-validation evaluation setting. Within each box plot, the mean and the median classification performance are depicted respectively with a dot and a horizontal line. **(A)** *T*_0_*vs*.*T*_4_, **(B)** *T*_1_*vs*.*T*_4_, **(C)** 5 classes.

**Figure 8:**
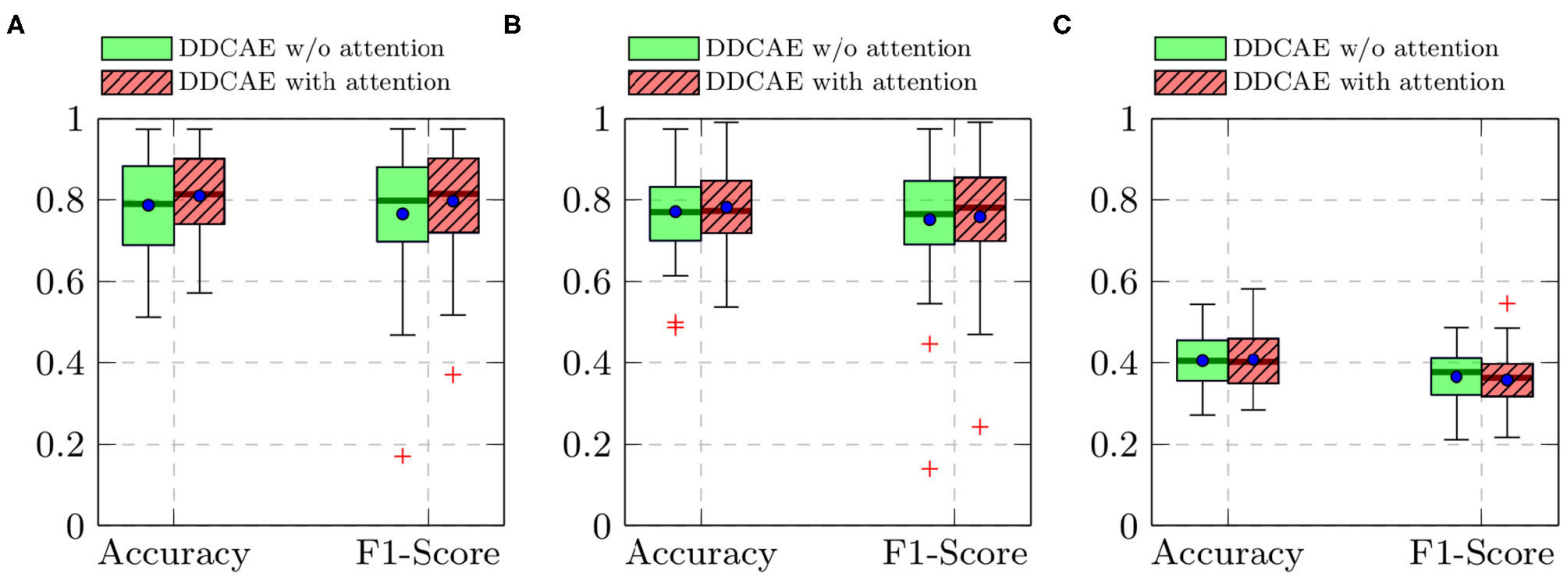
SenseEmotion Database: classification performance comparison in a *Leave One Subject Out* (LOSO) cross-validation evaluation setting. Within each box plot, the mean and the median classification performance are depicted, respectively with a dot and a horizontal line. **(A)** *T*_0_*vs*.*T*_3_, **(B)** *T*_1_*vs*.*T*_3_, **(C)** 4 classes.

Furthermore, the performance of the jointly trained classification model for each classification task is depicted in Figure 7 for the BioVid Heat Pain Database and in Figure 8 for the SenseEmotion Database. Moreover, a summary of the classification results is provided in Table 4. Concerning the BioVid Heat Pain Database, the proposed attention mechanism improves the performance of the multi-modal DDCAE across all classification tasks. The performance improvement is also significant in most cases in terms of F1-score. Concerning the SenseEmotion Database, the attention mechanism significantly improves the performance of the proposed approach regarding the binary classification task *T*_0_*vs*.*T*_3_.

**Table 4:** Classification performance comparison in a *Leave One Subject Out* (LOSO) cross-validation evaluation setting: Average Performance in % (Standard Deviation in %).

**BioVid Heat Pain Database (Part A)**						
Task	*T*_0_*vs*.*T*_4_		*T*_1_*vs*.*T*_4_		*T*_0_*vs*.*T*_1_*vs*.*T*_2_*vs*.*T*_3_*vs*.*T*_4_ (5Classes)	
Model	DDCAE		DDCAE		DDCAE	
	W/o attention	With attention	W/o attention	With attention	W/o attention	With attention
Accuracy	83.99(15.58)	**84.25(13.82)**	76.29(16.09)	**76.81(15.08)**	33.31(09.35)	35.44(08.66)^*^
F1-Score	78.66(25.43)	81.48(20.55)^*^	68.18(29.08)	71.92(24.81)^*^	30.31(09.30)	**31.04(07.91)**
**SenseEmotion Database**						
Task	*T*_0_*vs*.*T*_3_		*T*_1_*vs*.*T*_3_		*T*_0_*vs*.*T*_1_*vs*.*T*_2_*vs*.*T*_3_ (4Classes)	
Model	DDCAE		DDCAE		DDCAE	
	W/o attention	With attention	W/o attention	With attention	W/o attention	With attention
Accuracy	78.76(11.37)	81.05(10.73)^*^	77.19(11.70)	**78.26(10.21)**	40.48(06.88)	**40.77(07.00)**
F1-Score	76.64(15.99)	79.78(13.30)^*^	75.26(15.70)	**75.95(14.71)**	**36.53(06.61)**	35.75(06.69)

The best achieved performance is depicted in bold. An asterisk (*) depicts a significant performance improvement. The significance test is performed using a Two-Sided Wilcoxon-Signed-Rank test with a significance level of 5%.

In the case of the binary classification task *T*_1_*vs*.*T*_3_, the attention mechanism also improves the overall performance of the proposed architecture. In the case of the multi-class classification task (*T*_0_*vs*.*T*_1_*vs*.*T*_2_*vs*.*T*_3_), the improvement of the performance can only be seen in terms of accuracy. Overall, the proposed attention mechanism improves the performance of the designed multi-modal DDCAE across all classification tasks. Moreover, the depicted results show that applying a gated approach for the generation of a single weighted latent representation is not only beneficial for the reduction of the dimensionality of the final representation, but also, due to the optimized weighting parameters, the generated representation can significantly improve the performance of the classification system. In summary, the proposed gating layer is able to successfully perform an aggregation of the latent representations specific to each of the modalities and the resulting aggregated representation can be jointly used to optimize an effective inference model. Moreover, the proposed attention mechanism is able to improve the overall performance of the proposed multi-modal DDCAE model in terms of classification accuracy as well as reconstruction MSE. In the following sections, if not mentioned otherwise, the experiments are carried out with a version of the multi-modal DDCAE extended with the proposed attention mechanism.

### 4.3. Self-Supervised Approach: Results

To evaluate our self-supervised learning (SSL) algorithm we perform experiments on two datasets in the self-supervised setting: the BioVid Heat Pain Database (Part A) (Walter et al., 2013) (see Table 5) and the SenseEmotion Database (Velana et al., 2017; Thiam et al., 2019b) (see Table 6). In all our SSL experiments we kept the architecture as described in Table 1, with the exception of the output layer of the encoder network, where we have 512 units [256 for the mean and 256 for the log-variance of *p*(*h*|*x*)].

**Table 5:** Results for the BioVid dataset in the binary, five classes, and regression setting in a *Leave One Subject Out* (LOSO) cross-validation evaluation setting: average performance in % (standard deviation in %).

**BioVid Heat Pain Database (Part A)**						
**Task**	***T*_**0**_***vs***.***T***_**4**_**					
**Model**	**DDCVAE**			**DDCAE**		
**VAE prior**	**Adaptive**			**Fixed**	**N/A**	**N/A**
**method**	**Orig. data**	**Aug. data**	**Self-trained**	**self-trained**	**Aug. data**	**Orig. data**
**Samples**	3,440	30,960	3,440 + 860	3,440 + 860	30,960	3,440
**Accuracy**	69.3 (14.7)	84.0 (15.2)	83.0 (15.9)^†^	76.1 (16.8)	**84.2 (13.7)^†^**	69.0 (15.0)
**F1-Score**	63.8 (22.4)	80.0 (23.0)	78.0 (24.5)	65.8 (28.9)	81.5 (20.0)^*^	63.6 (22.9)
**Task**	***T*_0_*vs*.*T*_1_*vs*.*T*_2_*vs*.*T*_3_*vs*.*T*_4_**					
**Model**	**DDCVAE**			**DDCAE**		
**VAE prior**	**Adaptive**			**Fixed**	**N/A**	**N/A**
**method**	**Orig. data**	**Aug. data**	**Self-trained**	**self-trained**	**Aug. data**	**Orig. data**
**Samples**	8,600	77,400	8,600 + 2,150	8,600 + 2,150	77,400	8,600
**Accuracy**	25.8 (5.0)	**35.5 (7.9)**	32.9 (6.8)	29.0 (6.9)	35.4 (8.6)^*^	25.1 (5.5)
**F1-Score**	15.8 (5.0)	**31.6 (7.5)**	20.0 (6.0)	16.9 (5.7)	31.0 (7.9)^*^	17.5 (5.6)
**Task**	**Regression**					
**Model**	**DDCVAE**			**DDCAE**		
**VAE prior**	**Adaptive**			**Fixed**	**N/A**	**N/A**
**Method**	**Orig. data**	**Aug. data**	**Self-trained**	**self-trained**	**Aug. data**	**Orig. data**
**Samples**	8,600	77,400	8,600 + 860	8,600 + 860	77,400	8,600
**MAE**	1.21 (0.08)	0.97 (0.18)	1.00 (0.18)	1.03 (0.18)	0.97 (0.19)^*^	0.99 (0.21)
**RMSE**	1.41 (0.09)	1.16 (0.20)	1.18 (0.20)^†^	1.20 (0.16)	**1.16 (0.21)^†^**	1.17 (0.18)

† *p-Value of two-sided Wilcoxon-Signed-Rank Test is not signifying a statistically significant difference*.

* *p-Value of one-sided W-Test is signifying a statistically significant difference. For classification the alternative*.

*is “greater” and for regression “less”*.

*Bold font signifies the best result. The Wilcoxon-Signed-Rank tests have been carried out between the results of the self-supervised fine-tuning approach using only the original dataset (“DDCVAE Adaptive Prior Self-Trained”) and the DDCAE approach using the augmented dataset (“DDCAE Aug. Data”)*.

**Table 6:** Results for the SenseEmotion dataset in the binary, four classes, and regression setting in a *Leave One Subject Out* (LOSO) cross-validation evaluation setting: average performance in % (standard deviation in %).

**SenseEmotion Database**						
**Task**	***T*_0_*vs*.*T*_3_**					
**Model**	**DDCVAE**			**DDCAE**		
**VAE prior**	**Adaptive**			**Fixed**	**N/A**	**N/A**
**method**	**Orig. data**	**Aug. data**	**Self-trained**	**self-trained**	**Aug. data**	**Orig. data**
**Samples**	4,680	42,120	4,680 + 1,170	4,680 + 1,170	42,120	4,680
**Accuracy**	77.7 (11.3)	79.0 (11.0)	78.5 (11.4)	68.1 (11.8)	81.0 (10.7)^*^	52.3 (4.9)
**F1-Score**	74.2 (16.4)	75.5 (15.9)	73.8 (17.9)	51.8 (22.6)	79.8 (13.3)^*^	65.4 (7.9)
**Task**	***T*_0_*vs*.*T*_1_*vs*.*T*_2_*vs*.*T*_3_**					
**Model**	**DDCVAE**			**DDCAE**		
**VAE prior**	**Adaptive**			**Fixed**	**N/A**	**N/A**
**method**	**Orig. data**	**Aug. data**	**Self-trained**	**self-trained**	**Aug. data**	**Orig. data**
**Samples**	9,314	83,835	9,314 + 2,328	9,314 + 2,328	83,835	9,314
**Accuracy**	38.4 (6.6)	40.1 (6.5)	39.1 (5.9)	34.8 (6.3)	40.8 (7.0)^*^	31.5 (6.8)
**F1-Score**	30.0 (5.4)	36.6 (5.6)	27.4 (4.5)	23.3 (6.1)	35.8 (6.7)^*^	21.3 (10.8)
**Task**	**Regression**					
**Model**	**DDCVAE**			**DDCAE**		
**Prior**	**Adaptive**			**Fixed**	**N/A**	**N/A**
**method**	**Orig. data**	**Aug. data**	**Self-trained**	**self-trained**	**Aug. data**	**Orig. data**
Samples	9,314	83,835	9,314 + 2,328	9,314 + 2,328	83,835	9,314
MAE	0.82 (0.10)	0.80 (0.10)	0.81 (0.11)	0.85 (0.09)	0.80 (0.10)^*^	1.04 (0.07)
RMSE	0.97 (0.11)	0.96 (0.11)	0.96 (0.12)	1.01 (0.11)	0.96 (0.11)^*^	1.18 (0.09)

†*p-value of two-sided Wilcoxon-Signed-Rank Test is not signifying a statistically significant difference*.

* *p-value of one-sided W-Test is signifying a statistically significant difference. For classification the alternative*.

*hypothesis is “greater” and for regression “less”*.

*The Wilcoxon-Signed-Rank tests have been carried out between the results of the self-supervised fine-tuning approach using only the original dataset (“DDCVAE adaptive prior self-trained”) and the DDCAE approach using the augmented dataset (“DDCAE Aug. Data”)*.

We designed the experiments to investigate the influence of our self-supervised tuning method in combination with adaptive variational Auto-Encoders on the number of training samples required. To this end, we evaluate our approach with an adaptive prior as described in section 3 and with a fixed prior, i.e., a standard normal distribution as *p*(*h*). We evaluate the adaptive prior approach on the original data, on the augmented data (see section 3.5 for details) and in a self-supervised learning scenario, while we show results for the fixed prior on the augmented dataset. In all settings we trained the model for 100 epochs on the fully augmented data and evaluated on test data [*Leave One Subject Out* (LOSO) cross-validation evaluation]. For the self-trained setting, we trained the model on non-augmented data for 100 epochs, generated 25% additional data using the learned variational priors and classified them using model predictions, and fine tuned the previously trained model for 100 additional epochs using *only* the generated data. In the case of regression experiments, the performance measures are both Mean Absolute Error (MAE) and Root Mean Square Error (RMSE), defined as follows:

(20) $$\begin{array}{l} \text{MAE}=\frac{1}{T}\overset{T}{\underset{j=1}{\sum }}|y_{j}-\hat{y_{j}}| \end{array}$$

(21) $$\begin{array}{l} \text{RMSE}=\sqrt{\frac{1}{T}\overset{T}{\underset{j=1}{\sum }}{\left(y_{j}-\hat{y_{j}}\right)}^{2}} \end{array}$$

where *y*_*j*_ is the ground-truth class value of the *j*^*th*^ sample, $\hat{y_{j}}$ is the corresponding regression output value, and T is the number of samples. In all self-supervised fine-tuning experiments we used β_1_ = 0.001, β_2_ = 0.001, β_3_ = 0.001, λ = 0.9995. All Gaussian distributions were assumed to be isotropic, i.e., independent dimensions, which allows us to learn only the diagonal of the covariance matrices instead of the full matrix. We compute the variance of the Gaussian priors via the soft-plus activation of the corresponding output of encoder network, defined by *softplus*(*x*) = log[1 + exp(*x*)].

In all three settings (binary classification, all-vs.-all, and regression) we were able to achieve results that are only marginally lower compared to the fully augmented dataset, while only using a fraction of the samples (see column labeled “Self-Trained” of Tables 5, 6), thus showing the effectiveness of our method. All classification results were in the margin of 1–3% while only requiring an additional 25% of data points. The performance in terms of accuracy concerning the binary classification task of the BioVid Heat Pain Database was not significantly worse compared to the fully augmented case. Importantly, this does not hold for the fixed prior: the performances drop significantly compared to adaptive priors. We argue that this shows that our self-supervised approach enables the system to learn a informative representation for each of the modalities. Additionally, through the coupling introduced by the information-processing constraints, these representations are tuned in such a way that they improve the overall classification and regression performance, by discovering regularities in the data that can be exploited efficiently.

## 5. Discussion

We introduced and evaluated a novel approach to deep multi-modal pain intensity assessment. We evaluated our approach on two complex pain intensity assessment datasets and were able to achieve results comparable to current state-of-the-art methods (see Table 7). The results specific to the proposed multi-modal DDCAE architecture show that the joint optimization of a single latent representation for each specific input channel and a gating layer (with trainable parameters) to generate a weighted latent representation (that is subsequently fed into a jointly trained model to perform an inference task), can improve the overall performance of an entire architecture by multiple percent. Additionally the reconstruction of the input signals is also performed at a satisfactory extent. Furthermore, when combined with an appropriate attention mechanism, the performance of the entire architecture can be further significantly improved. Therefore, feature learning can be considered as a sound alternative to manual feature engineering, since the designed architecture is able to autonomously generate a set of relevant parameters without the need of expert knowledge in this particular area of application. As potential future work, an investigation of the temporal aspect of the physiological signals through the introduction of recurrent neural networks such as LSTMs should be undertaken. Moreover, since we set most of the hyper-parameters involved in the performed assessment of the proposed approaches empirically, methods designed to perform the fine-tuning of such hyper-parameters (Feurer and Hutter, 2019) may automate this step. The introduction and investigation of generative models [such as Generative Adversarial Networks (GANs)] for data augmentation in the case of bio-physiological data should also be undertaken and a comparison of the performances achieved by such approaches, with those achieved through SSL approaches could provide further insights into the dynamics involved in multi-modal inference tasks.

**Table 7:** Classification performance comparison with previous works (BioVid Heat Pain Database: *T*_0_ *vs*. *T*_4_).

**BioVid heat pain database (Part A)**	
**Approach**	**Accuracy (%)**
Early fusion with random forests (Werner et al., 2014)	74.10
Multi-modal DDCAE with a shared latent representation (Thiam et al., 2020a)	76.90
Multi-modal DDCAE with a concatenated latent representation (Thiam et al., 2020a)	77.24
Early fusion with random forests (Kächele et al., 2016, 2017)	82.73
Deep neural network ensemble with a weighted aggregation layer (Thiam et al., 2019a)	84.40
Multi-modal DDCAE with a gated latent representation (w/o attention)	83.99
Multi-modal DDCAE with a gated latent representation (with attention)	84.25
Multi-modal DDCVAE with a gated latent representation & SSL	83.00

*The performances of the proposed multi-modal DDCAE approaches are compared to other information fusion architectures involving the exact same modalities with an evaluation performed in a Leave One Subject Out (LOSO) cross-validation evaluation setting*.

In the Self-Supervised Learning setting we showed that our approach is able to drastically reduce the required number of training samples compared to classic data augmentation techniques. We achieved this by training a Deep Denoising Convolutional Adaptive Variational Auto-Encoder on each modality during a primary training period. We then use the learned latent prior for each modality to artificially generate new data samples, classify them with hard labels and perform a second fine-tuning training phase. Our method requires only 25% additional data with only a small performance loss. In an ablation study we were able to show that our adaptive VAE outperforms a classic VAE with a fixed prior, indicating that the additional flexibility allows to learn disentangled representations [encoded by their prior *p*(*h*)] for each modality. Given that we are working with temporal data it could be a promising research direction to investigate recurrent neural networks in the representation learning part, as these are known to be able to extract temporal dependencies in data. Furthermore, our self-supervised fine-tuning approach is independent of the underlying problem structure and we can therefore apply it to a variety of learning tasks. As potential future work one could investigate our method in the reinforcement learning setting to improve sample efficiency. A drawback of our method is that it requires careful tuning of the hyper-parameters β_1_, β_2_, and β_3_, as they have a drastic impact on the results. Chosen too small, the posterior can never diverge from the prior and thus no learning is possible, while a large value leads to large divergence and thus rendering the prior useless, as it does not capture the posterior. Meta-learning techniques may prove useful to automatically tune these hyper-parameters.

## 6. Conclusion

Even though the results depicted in the current work are very promising, pain recognition remains a very complex inference task. Several parameters have to be taken in consideration in order to ensure the effectiveness of the developed approaches. In the current work, the assessment of the proposed approaches is performed on data sets characterized by thermal pain elicitations. However, the outcome of the performed experiments can be biased by both the nature of the stimuli applied and the types of sensors used to perform the recordings. An assessment of the proposed approaches in diverse settings, using different types of painful stimuli such as pressure, cold or electrical stimuli, should therefore be conducted. Moreover, both data sets were recorded in controlled environments. Hence, the implementation and evaluation of the proposed approaches in real world settings would provide valuable insights and bring the whole research community one step further toward the goal of autonomously and effectively performing the assessment of different levels of pain. Such a technology would substantially improve the effectiveness of pain management in a clinical setting.

## Data Availability Statement

The original contributions presented in the study are included in the article/supplementary material, further inquiries can be directed to the corresponding author/s.

## Ethics Statement

The studies involving human participants were reviewed and approved by the ethics committee of the University of Ulm (Helmholtzstraße 20, 89081 Ulm, Germany) (ethical committee approval was granted: 196/10-UBB/bal). The patients/participants provided their written informed consent to participate in this study.

## Author Contributions

PT, FS, and HK conceived and designed the multi-modal DDCAE architecture. PT implemented and evaluated the architecture and collected and preprocessed the data used for the evaluation of the designed architectures. HH and DB designed the self-supervised architecture. HH implemented the self-supervised experiments and collected, interpreted, and evaluated the data. PT and HH interpreted and evaluated the results and wrote the first version of the manuscript. Funding Acquisition: FS, DB, and HK. All authors wrote, read, and approved the manuscript.

## Conflict of Interest

The authors declare that the research was conducted in the absence of any commercial or financial relationships that could be construed as a potential conflict of interest.

## Publisher's Note

All claims expressed in this article are solely those of the authors and do not necessarily represent those of their affiliated organizations, or those of the publisher, the editors and the reviewers. Any product that may be evaluated in this article, or claim that may be made by its manufacturer, is not guaranteed or endorsed by the publisher.
